# Remodeling of Cardiomyocytes: Study of Morphological Cellular Changes Preceding Symptomatic Ischemic Heart Failure

**DOI:** 10.3390/ijms241914557

**Published:** 2023-09-26

**Authors:** Milda Kuprytė, Vaiva Lesauskaitė, Vytenis Keturakis, Vitalija Bunevičienė, Lina Utkienė, Lina Jusienė, Dalia Pangonytė

**Affiliations:** 1 Laboratory of Cardiac Pathology, Institute of Cardiology, Lithuanian University of Health Sciences, LT-44307 Kaunas, Lithuania; milda.kupryte@lsmu.lt (M.K.); vytenis.keturakis@lsmuni.lt (V.K.);; 2 Laboratory of Molecular Cardiology, Institute of Cardiology, Lithuanian University of Health Sciences, LT-44307 Kaunas, Lithuania; vaiva.lesauskaite@lsmuni.lt

**Keywords:** remodeling, cardiomyocyte, histology, ischemia, heart failure

## Abstract

Although major pathogenesis mechanisms of heart failure (HF) are well established, the significance of early (mal)adaptive structural changes of cardiomyocytes preceding symptomatic ischemic HF remains ambiguous. The aim of this study is to present the morphological characterization of changes in cardiomyocytes and their reorganization of intermediate filaments during remodeling preceding symptomatic ischemic HF in an adult human heart. A total of 84 myocardial tissue samples from middle-left heart ventricular segments were analyzed histomorphometrically and immunohistochemically, observing the cardiomyocyte’s size, shape, and desmin expression changes in the remodeling process: Stage A of HF, Stage B of HF, and Stages C/D of HF groups (ACC/AHA classification). Values *p* < 0.05 were considered significant. The cellular length, diameter, and volume of Stage A of HF increased predominantly by the diameter vs. the control group (*p* < 0.001) and continued to increase in Stage B of HF in a similar pattern (*p* < 0.001), increasing even more in the C/D Stages of HF predominantly by length (*p* < 0.001). Desmin expression was increased in Stage A of HF vs. the control group (*p* < 0.001), whereas it was similar in Stages A and B of HF (*p* > 0.05), and most intense in Stages C/D of HF (*p* < 0.001). Significant morphological changes of cardiomyocytes and their cytoskeletal reorganization were observed during the earliest remodeling events preceding symptomatic ischemic HF.

## 1. Introduction

Heart failure (HF) is causing a significant clinical, public, and economic burden worldwide [1,2], remaining associated with frequent hospitalizations [3], high rates of in-hospital mortality, and rising global health expenditures [4]. Most cardiac muscle pathologies with predominating ischemic injuries contribute towards the progress of cardiac muscle dysfunction, which eventually manifests as symptomatic HF [5]. Although major pathogenesis mechanisms of HF are well established, the significance of early (mal)adaptive structural changes of cardiomyocytes preceding symptomatic ischemic HF remains ambiguous [6,7]. An ischemic injury initiates a complex of heterogenous compensatory molecular intracellular processes, which are meant to restore and maintain the regular function of the heart [8,9]. These compensatory processes, known as remodeling, manifest on intracellular and cellular levels, changing the inner cytoskeletal composition of intermediate filaments, such as desmin, interacting with sarcolemma, sarcomeres, and nucleus, and as a result, transforming the shape and improving the functional resilience of cardiomyocytes [10].

Continuous myocardial remodeling in the context of ischemic injury not only maintains cellular resilience under excessive muscle tension but also increases cellular energetic demands and disrupts the homeostasis of protein synthesis [8]. Desmin, as a predominating intermediate cytoskeletal filament, starts misfolding due to impaired post-translational modifications [11,12,13]. These events contribute to even more extensive damage of the heart muscle compared to the initial ischemic injury and become the structural basis for ischemic HF formation long before the first HF symptoms appear [8].

So far, data on early cardiomyocyte morphological changes occurring in remodeling and leading to HF are fragmented and controversial, based mostly on a quantitative organometric [14] or (semi)qualitative histological analysis of heart muscle tissue. Moreover, no substantial correlations between cellular size and inner cytoskeletal changes during the remodeling process of intermediate filaments, such as desmin, are based on extensive histomorphometric research. Usually, these studies present results based on low-quality selected samples [15], most of them exploring end-stage HF due to dilatative cardiomyopathy. Furthermore, ischemic injury is one of the predominating causes of HF, and by comparing tendencies of desmin expression in cardiomyocytes alone, some authors have already described significant differences between dilatative cardiomyopathies and heart muscle diseases with continuous ischemic injury. Additionally, most morphological data concerning HF pathogenesis come from animal-based studies with a rare focus on remodeling initiated and maintained by isolated continuous ischemic injury preceding symptomatic HF [16]. Therefore, knowledge about early structural changes in the remodeling of cardiomyocytes and their cytoskeletal reorganization specific to continuous ischemic injury in the human heart remains obscure.

This study is an attempt to present an extensive quantitative morphological characterization of the earliest cellular structural changes of cardiomyocytes that are observed during remodeling and precede symptomatic ischemic HF in an adult human heart. The characterization of remodeling in cardiomyocytes that suffer from continuous ischemic injury will provide substantial morphological evidence on the effect of remodeling for the pathogenesis of ischemic HF and will expand our overall knowledge of the complexity of the earliest cellular decompensation processes leading towards tissue deterioration and clinically diagnosed organ failure.

## 2. Results

### 2.1. Histomorphometric Analysis of Cardiomyocytes

Overall, 8638 cardiomyocytes from the middle segment of the left cardiac ventricle were fit for histomorphometric analysis via light microscopy according to the histomorphometric analysis criteria established in the study design. When analyzing two-dimensional cellular histomorphometric parameters (Table 1), the length of the cardiomyocytes in the longitudinal plane of Stage A of the HF group was already significantly increased compared to the control group (*p* < 0.001), indicating histomorphometrically detectable morphological cellular changes at the earliest stages of remodeling initiated by ischemic injury of the heart muscle and preceding symptomatic ischemic HF. According to further analysis of the cellular length, the value of this parameter in the cardiomyocytes of Stage B of the HF group was significantly increased compared to both cardiomyocytes in Stage A of the HF and control groups (*p* < 0.001), and the length of the cells in Stages C/D of HF was increased even more compared to those in Stage B of the HF group (*p* < 0.001).

Similar tendencies of cellular diameter changes were observed during the remodeling process when analyzing cardiomyocytes in Stage A of HF, Stage B of HF, and Stages C/D of the HF groups. A significant increase in the cellular diameter was already observed when comparing cardiomyocytes in Stage A of the HF group to the control group (*p* < 0.001). This histomorphometric parameter was increased in Stage B of the HF group compared to the same parameter in Stage A of the HF and control groups (*p* < 0.001). Also, the cellular diameter of the cardiomyocytes in Stages C/D of the HF group was increased compared to this parameter in Stage B of the HF group (*p* < 0.001).

Further analysis of the cellular volume confirmed similar tendencies of the morphological changes observed during the remodeling process in the cardiomyocytes. The cardiomyocytes in Stage A of the HF group were significantly larger compared to the control group (*p* < 0.001). The volume of cells in Stage B of the HF group significantly increased compared to the volume of cardiomyocytes in Stage A of the HF and control groups (*p* < 0.001). The cells in Stages C/D of the HF group were even larger compared to those in Stage B of the HF group (*p* < 0.001). 

Interestingly, when analyzing a length–diameter ratio of cardiomyocytes in Stage A of the HF, Stage B of the HF, and Stages C/D of the HF groups, more complex tendencies of cardiomyocyte remodeling were detected via cellular histomorphometric analysis. A significant decrease in the cellular length–diameter ratio was observed in Stage A of the HF group compared to the control group (*p* < 0.001), indicating the cellular growth pattern associated with predominantly increasing diameter over the length. Additionally, this cellular length–diameter ratio increased in Stage B of the HF group compared to Stage A of the HF group (*p* < 0.001). Yet, this value in Stage B of the HF group was smaller compared to the control group (*p* < 0.05). The cellular length–diameter ratio significantly increased in the cardiomyocytes of Stages C/D of the HF group compared to the cardiomyocytes in Stage A of the HF, Stage B of the HF, and the control groups (*p* < 0.001), indicating that the size of cardiomyocytes in this group increased mostly because of increasing cellular length compared to cellular diameter. 

### 2.2. Immunohistochemical Analysis of Desmin Expression in Cardiomyocytes

**Figure 1:**
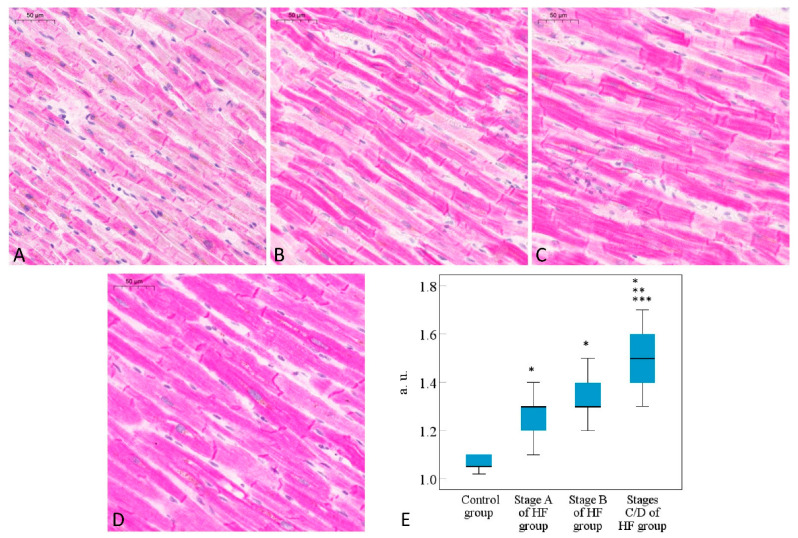
Desmin expression in the cardiomyocytes. Representative images of the myocardium immunohistochemistry in the control: (**A**) Stage A of the HF (**B**); Stage B of the HF (**C**); Stages C/D of the HF groups (**D**). Bar graph of the semi-quantitative analysis (**E**), results are presented as median, the 25th and 75th percentiles). * *p* < 0.001—statistically significant differences—Stage A of HF, Stage B of HF, Stages C/D of HF vs. the control group data; ** *p* < 0.001—Stage B of HF and Stages C/D of HF vs. Stage A of HF group data; *** *p* < 0.001—Stages C/D of HF vs. Stage B of HF (*Kruskal–Wallis’s* test with the pairwise comparisons were applied with the significance values adjusted by the *Bonferroni* correction for multiple tests). HF—heart failure; stages A, B, C, D of HF—according to ACC/AHA classification; a.u.—arbitrary units.

Overall preliminary observation of the results of an immunohistochemical reaction against the desmin in the selected heart muscle tissue samples was performed to detect the cardiomyocytes in the longitudinal plane that are fit for the study design to evaluate the tendencies of desmin expression in the cardiomyocytes (Figure 1).

Desmin expression in the cardiomyocytes evaluated using the immunohistochemical method already increased in Stage A of the HF group compared to the control group (*p* < 0.001), indicating the significant tendencies of desmin expression during the earliest stages of cardiomyocyte remodeling, preceding symptomatic ischemic HF. Desmin expression in the cardiomyocytes of Stage B of the HF group increased compared to the control group (*p* < 0.001), whereas there was no significant difference when comparing Stage B of the HF group to Stage A of the HF group (*p* > 0.05). Still, desmin expression was more significantly increased in Stages C/D of the HF group compared to Stage B of the HF, Stage A of the HF, and control groups (*p* < 0.001). Additionally, when observing desmin expression in the cardiomyocytes of Stages C/D of HF, cardiomyocytes with microscopically visible intrasarcoplasmic microaggregates of desmin were predominating, most likely indicating disrupted intracellular homeostasis of desmin in the late stages of remodeling. 

While exploring the associative trends of the desmin expression and the cellular histomorphometric parameters, statistically significant strong positive correlations were detected between intrasarcoplasmic desmin expression and cardiomyocyte length (r = 0.82, *p* < 0.001), diameter (r = 0.74, *p* < 0.001), and volume (r = 0.74, *p* < 0.001). The correlation defining the association between the cellular length–diameter ratio and the intracellular desmin expression was positively weak (r = 0.24, *p* < 0.05).

## 3. Discussion

The remodeling of the heart muscle is an active process of tissue reorganization, which is initiated in the context of cardiac tissue injury with the goal of maintaining an optimal pumping function by altering the composition of the tissue [17]. The tissues exposed to the injuring factors, such as continuous ischemia, constantly initiate compensatory remodeling changes, which are considered mostly positive in their effect to optimize inner homeostasis and the performance of specialized functions. Still, as protective and preserving these remodeling processes are considered, current data on the tissue remodeling processes indicate that, at some point, this remodeling becomes maladaptive, manifesting in reduced cardiac output and eventually leading to symptomatic HF [8]. On the other hand, some consider that these remodeling processes occurring in the heart muscle when exposed to a prolonged ischemic injury are pathological from the very beginning when these processes are initiated in the cardiomyocytes and may already indicate early heart muscle disease before the very first HF symptoms [5,17].

Our histomorphometric and immunohistochemical study of cardiomyocytes at different stages of ischemic HF according to the ACC/AHA classification presents novel extensive quantitative data on the cellular changes during the early and late phases of tissue remodeling, reporting the early disproportional changes in the cardiomyocytes preceding symptomatic ischemic HF. The cardiomyocytes already demonstrate significant changes in shape and intermediate filament desmin expression in the selected cases, representing an increased risk of developing HF without clinically identifiable myocardial structural disease. To the authors knowledge, this is the first detailed report with easily reproducible data characterizing the early and late remodeling events in cardiomyocytes on a cellular basis in an adult human heart before the very first symptoms of ischemic HF appear in a cohort of statistically representative size.

Increased overall volume and decreased length–diameter ratio of the cardiomyocytes in Stage A of the HF group compared to the control group indicates that the cardiomyocytes acquire morphological features of cellular hypertrophy with increased diameter over the cellular length at the earliest stages of the remodeling. According to these histomorphometric findings, it can be hypothesized that early remodeling with predominating increased cellular thickness due to the assembly of intracellular sarcomeric components mainly in parallel patterns occurs due to impaired contractility [18], manifesting in the early ischemic injuries of cardiac tissue. Further analysis of the histomorphometric data in Stage B of the HF (pre-HF) group detected a less disproportional increase in cardiomyocytes, with the cellular diameter changes being still predominant over cellular length, even in the late stages of remodeling preceding ischemic HF. This pattern of cardiomyocyte volume increase indicates that longer exposure to a continuous ischemic environment leads to decreased contractile elements that start transforming morphologic cellular features into those that macroscopically manifest as eccentric cardiac hypertrophy [19].

Hemodynamically, the purpose of these cellular changes is to induce cardiac hypertrophy as a compensatory response to reduce the tension of the cardiac ventricular wall and prevent cardiac dysfunction [8]. Still, a study on HF with preserved ejection fraction (HFpEF) reported by Mühlfeld C. et al. (2020) detected a significantly increased cellular diameter in early HF stage compared to a control group when applying the histomorphometric approach of tissue remodeling analysis to an animal heart [20], considering these morphological changes as a part of the structural basis for the already developing heart muscle disease. Interestingly, a study on idiopathic dilatative cardiomyopathy performed by Vigliano C. A. et al. (2011) identified that more than 50% of the patients with an increased mean diameter of their cardiomyocytes had significantly worse HF-associated outcomes, and the *Cox* regression univariate analysis determined increased cardiomyocyte diameter as an important independent predictor of patient’s death [21], further supporting the hypothesis that detected morphological changes in the remodeling preceding ischemic HF are considered part of myocardial disease from the very beginning. Altogether, these tendencies suggest that increased cardiomyocyte diameter may be disproportionally one of the first morphological changes of the remodeling in the context of ischemic injury when compared to cellular length, also demonstrating the ambiguous nature of these remodeling processes that represent not only the compensatory capacity of heart muscle but also become a predicting factor for symptomatic ischemic HF in the future. 

The early remodeling processes initiated in the cardiomyocytes preceding symptomatic ischemic HF promote significant increases in the overall cellular volume (and simultaneously, the mass of cardiac ventricle), supposedly strengthening the suggestion of contractility function being gradually lost as a result of ischemic injury, and thus increasing cardiac preload. Although these cellular events are considered to be compensatory in the context of ischemic injury, the morphological changes associated with the early remodeling of cardiomyocytes also lead to significant circumferential shortening of the cardiac ventricle in the remote regions of the previously observed ischemic injuries with reduced systolic wall movements and ejection fraction [22], further emphasizing a novel and more intricate role of early cardiomyocyte remodeling in the pathogenesis mechanism of symptomatic ischemic HF.

Despite the predominating compensatory aspect of early remodeling, these structural changes in the cardiomyocytes during the early stages of remodeling provide only a short-term effective solution to improve the contractile activity of heart muscle. The oxygen demand and perfusion of the myocardium are altered due to initiated hypertrophic changes when the capillary density is relatively decreased, contributing to the reinforcement of the pathology by accelerating cardiac contractile dysfunction [18]. Further results from the histomorphometric analysis of the cardiomyocytes in Stages C/D of the HF group, representing the morphological changes in the symptomatic ischemic HF, revealed that the maladaptive remodeling process continues to induce significant hypertrophic changes in the cardiomyocytes with an increasing cellular length over diameter, as well as an overall cellular volume. According to Tamura T. et al. (1998), the lengthening of cardiomyocytes alone accounts for increased cardiac chamber circumference progressing to HF [23]; therefore, significant changes in the longitudinal axis of the cardiomyocytes can be assumed as a histomorphological feature of decompensation. A study conducted by Janczewski A. M. et al. (2002) determined similar results through the use of the histomorphometric approach of a single left cardiac ventricle cardiomyocyte length in dilatative cardiomyopathy [24]. Interestingly, no significant differences in the cellular diameter were detected when comparing the experimental and the control groups in the same study, whereas our study determined a moderate but statistically significant increase in cardiomyocyte diameter in Stages C/D of the HF group, implying more complex morphological differences in remodeling in cases of continuous ischemic cardiac injury compared to dilatative cardiomyopathy.

In response to continuous ischemia, cardiomyocytes tend toward hypertrophy with predominant lengthening during late remodeling in order to maintain physiological stroke volume with a reduced number of properly functioning myocardial tissue segments [25]. Nonetheless, these cellular changes during cardiomyocyte remodeling result in overstretching, exhaustion of the compensatory Frank–Starling mechanism, ventricular dilatation [5], and increasing cardiac wall stress, which contribute to the progression of ventricular wall thinning [26], thus creating a vicious circle and further supporting the role of the adverse remodeling in the pathogenesis mechanism of the advancing ischemic HF.

In fact, results from studies on ischemic HF demonstrate that the morphological changes in cardiomyocytes induced by adverse remodeling contribute towards a clinically significant deterioration of the heart muscle more than any other structural component of the organ in cases of prolonged ischemia [27]. According to Del Buono M. G. et al. (2022), in response to continuous ischemic exposure, elongation of hypertrophic cardiomyocytes becomes a predominant factor, causing significant cardiac geometry changes, increasing the mass of the cardiac wall, and left ventricular enlargement, eventually contributing to increased systolic longitudinal wall stress and irreversible global ventricular dysfunction, manifesting as symptomatic HF [28]. Simultaneously, significant changes in hemodynamics and neurohormonal activation reflect the overall decreasing efficiency in the functionality of the cardiovascular system. A continuous reduction in cardiac output contributes towards the activation of the renin–angiotensin–aldosterone system and, as a result, affects the size and disturbs the metabolism of cardiomyocytes, also increasing protein synthesis in various different cells of the myocardial tissue, including fibroblasts, which tend to produce the extracellular matrix, thus promoting the increasing stiffness of the myocardial tissue and diastolic cardiac dysfunction [29].

As a result of the remodeling induced by continuous ischemic injury to the heart muscle, intrasarcoplasmic protein homeostasis is affected, and structural alterations of the cardiomyocyte cytoskeletal components also occur [30,31,32]. Desmin, being a major load-bearing structure of cardiomyocytes, as an intermediate filament, participates in ensuring cellular resilience against mechanical stressors, maintaining sarcomeric integrity, optimizing the intrasarcoplasmic communication between the different intracellular compartments and transmitting the signals of the cellular response towards the extracellular microenvironment [33]. Cells with desmin deficiency are not able to respond to extracellular stimuli by promoting compensatory reactions; therefore, these intracellular processes are disrupted and managed without a sufficiently balanced organization of intrasarcoplasmic compartments [34,35].

An immunohistochemical study of the desmin expression in the cardiomyocytes detected a significantly increased desmin expression in the cardiomyocytes of Stage A of HF and Stage B of HF compared to the control group, indicating the specific features of the early reorganization of the cytoskeletal components in cardiomyocytes during remodeling. Indeed, the upregulation of desmin expression in the early stages of any cardiac disease may be assumed to be a compensatory process [36] because no immunohistochemical features of desmin expression disarray are observed in these groups. A study conducted by Pawlak A. et al. (2012) presented similar tendencies in terms of desmin expression in the HF due to dilatative cardiomyopathy and noticed that the desmin expression increases significantly as HF develops [37]. Also, a study on ischemic HF by Bouvet M. et al. (2021) detected that an increasing amount of phosphorylated desmin impacts its solubilization and creates the intracellular conditions for irreversible aggregate formation in the future when the deterioration of the heart muscle is progressing towards symptomatic HF [38].

Yet, when comparing desmin expression in Stage A of the HF and Stage B of the HF groups, the expression of this intermediate filament is similar in both groups in terms of cardiomyocytes, and the immunohistochemical reaction preserves its striated cytoplasmic pattern in most cardiomyocytes. Therefore, one can assume that the cytoskeletal reorganization process is efficient in the early stages of remodeling, preserving the regular assembly mechanisms of the intermediate filaments and the overall integrity of the cytoskeletal structures. Interestingly, it may also be hypothesized that the undistorted desmin arrangement observed during the later events of the remodeling continues to mostly maintain the organized functional resilience and contractility of the cardiomyocytes [38], thus preventing the more rapid progression of heart muscle disease into the symptomatic HF to some level.

The increased expression of desmin with disrupted intrasarcoplasmic organization and microaggregates in the cardiomyocytes are observed predominantly in symptomatic ischemic HF, indicating that these morphological features represent end-stage remodeling processes in failing cardiomyocytes [39]. As a result of continuous ischemic injury and mechanical tension, the post-translational processing of desmin is performed more actively, creating different proteoforms of desmin typical to irreversibly dysfunctional heart muscle [35]. Also, the enhanced activity of the post-translational modifications or mutations of the N-terminal domain of desmin [40] may cause the misfolding of this intermediate filament, further disrupting the organizational compartments of sarcomeric components [34] and producing pro-amyloidogenic oligomers [12,40], which may become a source for protein aggregate formation, thus contributing to direct toxic cellular injury and altering the overall mechanics of the sarcomeric component of the cardiomyocytes in the symptomatic ischemic HF irreversibly [37,41].

Overall, the extensive characterization of the remodeling process in the cardiomyocytes preceding symptomatic ischemic HF based on histomorphometric and immunohistochemical analyses in a statistically representative cohort provides a structurally based insight into the dynamics of the remodeling process and its role in the pathogenesis of ischemic HF. Although the relevance of the remodeling process in the pathogenesis of HF is well recognized, practical translation of the structural data on the remodeling process into clinical, diagnostic settings remains controversial and problematic due to the poor reproducibility of these data [15]. Still, the role of using uniform reference criteria in morphological cardiac tissue assessment and thus maintaining efficient collaboration between clinicians, radiologists, molecular biologists, and geneticists is acknowledged [42]. Therefore, the knowledge of the cellular changes in the cardiomyocyte remodeling process based on the quantitative structural data obtained from the study with a representative cohort may provide a practical diagnostic solution, creating an objective, unbiased diagnostic tool as part of a clinical follow-up panel of the course of myocardial disease in the context of continuous ischemic injury, assisting in evaluating the effects of the medications improving cardiac remodeling processes, and providing structurally validated basic data for future studies of the remodeling process in cardiomyocytes.

## 4. Materials and Methods

### 4.1. Study Design and Groups

Myocardial tissue samples from the middle segments of the human left cardiac ventricles were selected from the paraffin blocks of the cardiovascular tissue collection of the Laboratory of Cardiac Pathology of the Institute of Cardiology (Lithuanian University of Health Sciences, LUHS, Kaunas, Lithuania) to achieve the goal of exploring the remodeling of cardiomyocytes by detecting the earliest morphological cellular changes preceding symptomatic ischemic HF.

Overall, 84 selected myocardial tissue samples were further classified into three groups according to the archival clinical data and the results of the histomorphologic analysis of the heart (Table 2).

The characterization of the groups selected to detect the morphological changes in cardiomyocyte remodeling preceding symptomatic ischemic HF:

- Stage A of HF (At risk for HF group)—patients who died suddenly within 1 h after the first clinical symptoms of myocardial infarction (MI) in the witnessed cases or within 24 h of last being seen alive in the unwitnessed cases [44,45]; no previous symptoms of HF were reported, no scars after MI were detected during the morphological tissue inspection, the acute ischemic injuries were up to 6 h [46], HF was diagnosed as being A stage according to the American College of Cardiology (ACC)/American Heart Association (AHA) classification [43], and an extensive morphological examination of the heart was performed during this postmortem procedure (*n* = 26);
- Stage B of HF (Pre-HF group)—patients who died suddenly due to the cardiovascular complications associated with the ischemic heart injury within 1 h after the first clinical symptoms in the witnessed cases or within 24 h of last being seen alive in the unwitnessed cases [44,45]; no previous symptoms of HF were reported, but a scar after MI was detected in the postmortem morphological inspection of the heart, HF was classified as B stage according to the ACC/AHA classification [43], and the extensive morphological examination of the heart was performed during this postmortem procedure (*n* = 25);
- Stages C/D of HF (Symptomatic/Advanced HF group)—patients who were diagnosed with symptomatic ischemic HF classified as C or D stage according to ACC/AHA classification [43], a heart transplantation procedure was performed for them, and an extensive morphological examination of surgical material after the failing heart procedure was carried out (*n* = 33).

Male patients who died from external causative factors or acute non-cardiovascular diseases with their hearts being examined during the postmortem procedure were selected as a control group (*n* = 25). 

No other disease that could cause the reorganization of the myocardial tissue components (e.g., systemic arterial hypertension, congenital or acquired cardiac valve disease, cardiomyopathy, diabetes mellitus, pulmonary diseases, etc.) was diagnosed for these patients.

The samples of the myocardial tissue from the middle segments of the free wall of the left cardiac ventricles in all three groups were obtained for further microscopic testing, avoiding areas of close MI or scarring after MI. Detailed histomorphological diagnostics were performed for all the selected cases before a histomorphometric study. 

### 4.2. Histomorphometric Analysis of Cardiomyocytes

A histomorphometric approach was adopted to objectively evaluate the remodeling changes in cardiomyocyte size and shape. Therefore, 5 formalin-fixed paraffin-embedded serial sections of 4 µm thickness were produced for each selected case, applying the stepwise approach with a distance of 20 µm to avoid the same overlapping cardiomyocytes being measured several times in each selected case. The produced serial sections were stained using the *Heidenhain’s azan trichrome* method to visualize the intercalated discs of the cardiomyocytes in the longitudinal plane required for histomorphometric cellular analysis. The histological slides stained using the *Heidenhain’s azan trichrome* method were examined with light microscopy (motorized microscope Olympus BX51, Olympus Corporation, Tokyo, Japan) at 20× magnification and 80 representative microscopic fields in 5 serial sections for each selected case in Stage A of HF, Stage B of HF, Stages C/D of HF, and the control groups were recorded by a digital camera (Evolution Qei, Media Cybernetics, Rockville, MD, USA) to collect and measure up to 80 cardiomyocytes for the each selected case.

The histomorphometric analysis of the stored, recorded images was performed using Image Pro Plus 6.0 software (Media Cybernetics, Rockville, MD, USA), measuring the two-dimensional cellular histomorphometric parameters (the length and the diameter), afterwards calculating the length–diameter ratio (cell length/cell diameter) and the volume ((π × (0.5 × cell diameter)^2^) × cell length) for each cardiomyocyte in the representative longitudinal plane. The cardiomyocytes in the longitudinal plane were considered representative when the intercalated discs at both lateral cellular tips and the central position of the nucleus were observed using light microscopy. The histomorphometric analysis was performed in a blind manner to avoid observer bias, and none of the researchers knew what groups of the selected cases during this process of histomorphometric analysis were.

### 4.3. Semi-Quantitative Immunohistochemical Analysis of Desmin Expression in Cardiomyocytes

An immunohistochemical approach was adopted to objectively evaluate the changes in intracellular intermediate filament desmin expression during the remodeling process. The selected formalin-fixed paraffin-embedded myocardial tissue samples of Stage A of HF (*n* = 26), Stage B of HF (*n* = 25), Stages C/D of HF (*n* = 33), and the control (*n* = 25) groups (see Table 2) were cut into 3 µm thick sections and placed onto SuperFrost slides (Menzel, Braunschweig, Germany). The deparaffinization sections were washed with distilled water, and the epitope retrieval procedure was performed using a microwave tissue processor RHS-1 (Milestone Medical, Roseland, NJ, USA) and incubating the samples in TRIS/EDTA buffer (Target Retrieval Solution, pH 9.0, Agilent Technologies Inc., Wood Dale, IL, USA) at 110 °C for 8 min. Immunohistochemical staining was performed using “Shandon Coverplate” plates (Thermo Fisher Scientific, Waltham, MA, USA). Endogenous peroxidase was blocked, and the samples were incubated with the primary rabbit polyclonal antibody against desmin (Cat#HPA018803, RRID: AB_1847616, Sigma-Aldrich, Merck Group, St. Louis, MO, USA), as validated by the *Human Protein Atlas* project, and diluted to 1:250 in the antibody diluent (Dako REAL, Agilent Technologies Inc., Wood Dale, IL, USA) for 1 h. The binding sites were visualized using the “EnVision Flex+” visualization system with the *HRP Magenda* chromogen (Agilent Technologies Inc., Wood Dale, IL, USA), counterstaining the sections with *Mayer’s* hematoxylin and coating with cover glasses using a polystyrene coating material. The tissues taken from an appendix served as a positive control, and tonsil fragments served as a negative control, applying the same protocol used for the immunohistochemical reaction as that used in the analyzed myocardial samples and running these reactions simultaneously. The IgG of the same isotype as the primary antibody dilution served as a reagent control.

The semi-quantitative analysis of the immunohistochemical reaction against desmin was performed using a score evaluation for the immunohistochemical reaction intensity and pattern: 1 point, physiological expression; 2 points, increased desmin expression with preserved intracytoplasmic striated pattern; and 3 points, increased desmin expression with deranged intracytoplasmic striated pattern, intracytoplasmic microaggregate formation. The results of the immunohistochemical reaction were observed in the 50 microscopic fields at 40× magnification (light microscopy, motorized microscope Olympus BX51, Olympus Corporation, Tokyo, Japan) for each produced microsection for each selected case, indicating the number of cardiomyocytes within each reaction pattern separately in the whole microsection area of the selected case according to the study design (% of all the longitudinal plane cardiomyocytes visible in the whole myocardial microsection). The immunohistochemical reaction was evaluated only in those cardiomyocytes where both the lateral cellular intercalated discs and the central nucleus were visible in the longitudinal plane. A formula to evaluate the overall scope of the intensity of the immunohistochemical reaction in the selected myocardial tissue was applied: ((1 point × count of the representative cardiomyocytes %) + (2 points × count of the representative cardiomyocytes %) + (3 points × count of the representative cardiomyocytes %))/100. The evaluation of the immunohistochemical reactions was presented in arbitrary units (a.u.). The evaluation was performed by the two researchers independently, and none of these researchers knew the groups of the selected cases during this evaluation process. The inter-observer and intra-observer variability was evaluated via Kappa (κ) statistics (*Cohen*’s κ coefficient > 0.9).

### 4.4. Statistical Analysis

The normality of the data distribution was assessed with the *Kolmogorov–Smirnov* tests. The numerical data that fit a normal distribution were reported as the mean (standard error, SE) with 95% confidence intervals (CI). The numerical data that did not fit a normal distribution were reported as the median with the interquartile range. The statistically significant differences between Stage A of HF, Stage B of HF, Stages C/D of HF, and the control groups were determined via ANOVA with post hoc *Bonferroni* tests for the multiple comparisons when the numerical data fitted the normal distribution. The nested design of the ANOVA was used to compare the numerical variables of the histomorphometric data, assuming Stage A of HF, Stage B of HF, Stages C/D of HF, and the control groups as a fixed factor and the histomorphometric measurements within each case as a random factor. When the numerical data did not fit the normality distribution, independent samples *Kruskal–Wallis*’s test with pairwise comparisons were applied to compare the results between Stage A of HF, Stage B of HF, Stages C/D of HF, and the control groups, also analyzing the significance values adjusted using *Bonferroni* correction for the multiple tests. *Spearman’s* rank correlation was applied to evaluate the correlation trends. The values of *p* < 0.05 were considered statistically significant. The statistical analysis was performed using the Statistical Package for the Social Sciences (SPSS) software (SPSS Statistics version 29.0, IBM, Armonk, NY, USA).

## 5. Conclusions

Overall, the significant morphological changes in the cardiomyocytes and their cytoskeletal reorganization are already observed during the earliest events of remodeling, when heart muscle is exposed to continuous ischemic injury, and these specific structural changes precede symptomatic ischemic HF. The morphological alterations of the cellular diameter and length correlating with the increased desmin expression during the ongoing remodeling are extensively characterized in this study, presenting quantitative morphological evidence of the early structural cellular and cytoskeletal changes, with a focus on their morphofunctional impact on the pathogenesis of symptomatic ischemic HF. The morphological features that are specific to cardiomyocytes during early remodeling preceding symptomatic ischemic HF may be applied to pathological diagnostic practices as part of histomorphometrically and immunohistochemically validated diagnostic tools to identify the earliest structural features of failing cardiomyocytes before the first symptoms of HF appear to optimize a physiologically guided and timely individualized treatment strategy.

## Figures and Tables

**Table 1:** Histomorphometric parameters of cardiomyocytes in stage A of HF, stage B of HF, stages C/D of HF, and the control groups.

Parameter	Control Group	Stage A of the HF Group	Stage B of the HF Group	Stages C/D of the HF Group
Number of the representative cardiomyocytes	1929	2080	1992	2637
Mean length (SE), µm	61.82 (0.34)	72.23 * (0.32)	78.86 *^,^** (0.33)	103.28 *^,^**^,^*** (0.29)
95% CI of length, µm	61.16–62.47	71.59–72.86	78.21–79.50	102.71–103.83
Mean diameter (SE), µm	11.73 (0.06)	14.34 * (0.05)	15.19 *^,^** (0.05)	18.92 *^,^**^,^*** (0.05)
95% CI of diameter, µm	11.62–11.84	14.23–14.45	15.08–15.30	18.83–19.02
Mean volume (SE), µm^3^	7271 (201)	12,320 * (193)	15,170 *^,^** (197)	31,433 *^,^**^,^*** (172)
95% CI of volume, µm^3^	6877–7666	11,941–12,699	14,783–15,557	31,096–31,769
Mean cellular length–diameter ratio (SE)	5.392 (0.025)	5.137 * (0.024)	5.301 ^#,^** (0.025)	5.583 *^,^**^,^*** (0.022)
95% CI of cellular length–diameter ratio	5.343–5.442	5.090–5.185	5.252–5.349	5.541–5.625

All multiple comparisons were performed by applying the nested design of ANOVA with *Bonferroni* post hoc tests: * *p* < 0.001—Stage A of HF, Stage B of HF, Stages C/D of HF vs. control group; ** *p* < 0.001—Stage B of HF and Stages C/D of HF vs. HF of A group; *** *p* < 0.001—HF of C/D stage vs. Stage B of HF; ^#^ *p* < 0.05—Stage B of HF group vs. control group. HF—heart failure; stages A, B, C, D of HF—according to ACC/AHA classification; SE—standard error; CI—confidence interval.

**Table 2:** Characterization of the groups selected to detect the morphological changes in cardiomyocyte remodeling preceding symptomatic ischemic HF.

	Control Group	Stage A of the HF Group	Stage B of the HF Group	Stages C/D of the HF Group
Number of cases	25	26	25	33
Mean age (SD), years	50.5 (8.7)	54.4 (8.6)	54.4 (7.4)	56.8 (7.5)
Sex	Male	Male	Male	Male
Cardiovascular disease of ischemic origin	No	Yes	Yes	Yes
Previous clinical symptoms of HF	No	No	No	Yes
Stage of HF according to ACC/AHA *	Not applied	At-Risk for HF	Pre-HF	Symptomatic HF

* Stage of HF was diagnosed according to the American College of Cardiology (ACC)/American Heart Association (AHA) classification [43]. HF—heart failure; stages A, B, C, D of HF—according to the ACC/AHA classification; SD—standard deviation.

## Data Availability

The data presented in this study are available from the corresponding author upon reasonable request.
